# *Aesculus hippocastanum* extract and the main bioactive constituent β-escin as antivirals agents against coronaviruses, including SARS-CoV-2

**DOI:** 10.1038/s41598-024-56759-y

**Published:** 2024-03-17

**Authors:** Freddy Armando Peñaranda Figueredo, Josefina Vicente, Andrea Alejandra Barquero, Carlos Alberto Bueno

**Affiliations:** 1 Laboratorio de Virología, Departamento de Química Biológica, Facultad de Ciencias Exactas y Naturales, Universidad de Buenos Aires, Buenos Aires, Argentina; 2 Instituto de Química Biológica de la Facultad de Ciencias Exactas y Naturales (IQUIBICEN), CONICET-Universidad de Buenos Aires, C-1428GBA Buenos Aires, Argentina

**Keywords:** Drug discovery, SARS-CoV-2, Virology, Antivirals, Antimicrobial responses, Viral infection

## Abstract

Respiratory viruses can cause life-threatening illnesses. The focus of treatment is on supportive therapies and direct antivirals. However, antivirals may cause resistance by exerting selective pressure. Modulating the host response has emerged as a viable therapeutic approach for treating respiratory infections. Additionally, considering the probable future respiratory virus outbreaks emphasizes the need for broad-spectrum therapies to be prepared for the next pandemics. One of the principal bioactive constituents found in the seed extract of *Aesculus hippocastanum* L. (AH) is β-escin. The clinical therapeutic role of β-escin and AH has been associated with their anti-inflammatory effects. Regarding their mechanism of action, we and others have shown that β-escin and AH affect NF-κB signaling. Furthermore, we have reported the virucidal and broad-spectrum antiviral properties of β-escin and AH against enveloped viruses such as RSV, in vitro and in vivo. In this study, we demonstrate that β-escin and AH have antiviral and virucidal activities against SARS-CoV-2 and CCoV, revealing broad-spectrum antiviral activity against coronaviruses. Likewise, they exhibited NF-κB and cytokine modulating activities in epithelial and macrophage cell lines infected with coronaviruses in vitro. Hence, β-escin and AH are promising broad-spectrum antiviral, immunomodulatory, and virucidal drugs against coronaviruses and respiratory viruses, including SARS-CoV-2.

## Introduction

Respiratory viruses can cause severe, life-threatening illnesses, such as sepsis and acute respiratory distress syndrome (ARDS). The focus of treatment for such viruses is on supportive therapies and direct antivirals to prevent virus replication and dissemination in the host. In the management of influenza, effective antiviral options include neuraminidase inhibitors, namely oseltamivir, zanamivir, and peramivir. Meanwhile, for SARS-CoV-2, therapeutic agents include ritonavir-boosted nirmatrelvir (Paxlovid), remdesivir, and molnupiravir. However, antiviral drugs may cause resistance by exerting selective pressure on the pathogen^1,2^. Despite the differences in host response to viral infections, the downstream effects are often the same, resulting in excessive inflammation and a loss of lung barrier integrity, which can lead to oedema and tissue damage^1^. Modulating the host response has emerged as a viable therapeutic approach for treating respiratory infections. The recent COVID-19 pandemic has made clear that this approach may be valuable, as treatment with corticosteroids such as dexamethasone has a significant impact on outcomes^1,3^.

One of the principal bioactive constituents found in the seed extract of *Aesculus hippocastanum* L. (AH) is β-escin. The clinical therapeutic role of β-escin and AH has been associated with their anti-inflammatory effects^4,5^. Regarding their mechanism of action, we and others have shown that β-escin and AH affect NF-κB signaling in various cell types and conditions in vitro^6–8^. In addition, we have reported the virucidal and broad-spectrum antiviral properties of β-escin and AH against the enveloped viruses HSV-1, VSV, RSV, and DENV in vitro*.* Recently, Lai et al. have described the inhibitory effect of β‑escin on Zika virus infection, noting a tenfold reduction in viral titers and nearly a 98% reduction in viral RNA levels observed with a concentration of 30 µM of β-escin in Vero cells^7–9^. Interestingly, AH treatment improves the course of acute disease in a murine model of pulmonary RSV infection, as evidenced by decreased weight loss, and reduced RSV lung titers and airway inflammation^8^.

Thus, considering the well-known anti-inflammatory effects of β-escin and AH in clinical therapy, and their virucidal and broad-spectrum antiviral activities against enveloped viruses, the aim of the current study was to expand the antiviral activity of β-escin and AH to coronaviruses, as well as to evaluate their effect on the activation of the NF-κB signaling pathways and on the production of different cytokines in cells infected with coronaviruses. As there may be disparities in the biological responses to β-escin and AH, we deem it crucial to analyze the effects of both treatments.

## Materials and methods

### Reagents

β-escin and AH were kindly provided by Spedrog Callion S.A., Buenos Aires, Argentina. All methods were carried out in accordance with relevant institutional, national, and international guidelines and legislation. β-escin was originally purchased from Indena S.p.A., Milan, Italy (Batch N° 31259/M2; purity of 98.3%), and AH from Martin Bauer Group (Finzelberg GmbH & Co), Andernach, Germany (Batch N° 13013823). The identification and purity were analyzed by Spedrog Callion S.A. and complies with the specifications of the Argentinian Pharmacopoeia. AH was dissolved in Minimal Essential Medium (MEM) without supplementation, and β-escin was dissolved in dimethylsulfoxide (DMSO) and diluted with MEM. The maximum concentration of DMSO used (1%) exhibited no toxicity under in vitro conditions. The rabbit polyclonal antibody anti-Spike of SARS-CoV-2 was obtained from Sigma-Aldrich, USA. Secondary goat anti-rabbit FluoroLinkTMCyTM2 antibodies were purchased from GE Healthcare, USA. DAPI was purchased from Sigma-Aldrich.

### Cells and viruses

Murine macrophage cell line J774A.1 was kindly provided by Dr. Osvaldo Zabal (INTA–Castelar, Buenos Aires, Argentina) and grown in MEM supplemented with 10% inactivated fetal bovine serum (FBS). Vero E6 cells and CRFK cells were grown in MEM supplemented with 10% FBS. Vero E6 and SARS-CoV-2 Wuhan strain were kindly provided by Dr. Jorge Quarleri (INBIRS-Buenos Aires, Argentina). Canine coronavirus (CCoV) and CRFK cells were kindly provided by Dr. Carlos Palacios (Fundación Pablo Cassará-Buenos Aires, Argentina). Calu-3 cells were kindly provided by Dr. Fernanda Elias (Fundación Pablo Cassará-Buenos Aires, Argentina), and calu-3 cells were grown in D-MEM supplemented with 10% FBS. CCoV and SARS-CoV-2 were used and propagated at low multiplicity of infection (moi).

### Antiviral activity

Virus yield inhibition assay was performed as previously described^7,8,10^. Briefly, cells grown in 24-well plates were infected with a moi of 0.1. Following 1 h of adsorption at 37 °C, the inoculum was removed, and cells were exposed to the compounds during 24 h. Supernatants were then collected and titrated by plaque assay, and the concentration required to reduce the virus yield by 50% (EC_50_) was calculated.

### Virucidal effect

CCoV and SARS-CoV-2 (10^7^ PFU) were diluted in culture medium containing or not each compound and incubated for 120 min at 37 °C. Aliquots were diluted to a non-inhibitory drug concentration and titrated by plaque assay on CRFK and Vero E6 cells, respectively.

### Time-of-addition assays

These assays were performed as previously described with minor modifications^7,8^. In brief, for pre-infection assays, cells were treated with the compounds for 2 h at 37 °C, washed with PBS and then infected with CCoV (moi = 0.1). For co-infection, cells were simultaneously infected with CCoV and treated with the respective compound. After 1 h of adsorption at 37 °C, the virus-drug mixture was removed, washed and a compound free medium was added. For post-infection (p.i.) assays, cells were infected with CCoV for 1 h at 37 °C and then treated with the tested compound at 0, 2, 4, 6, 8, 12 and 16 h after infection. A control culture that was infected but not treated (CV) was simultaneously studied. Cells were further incubated at 37 °C for up to 24 h p.i., and after cell disruption by means of freezing and thawing, supernatants were titrated by plaque assay in CRFK cells.

### Cytotoxicity assay

Cells grown in 96 well plates were treated with different concentrations of β-escin and AH for 24 h. Mock was considered as 100% of cell viability. Finally, samples were analyzed using the 3-(4,5-dimethylthiazol-2-yl)-2,5-diphenyltetrazolium bromide (MTT) (Sigma-Aldrich) assay, following the manufacturer's instructions.

### Adsorption assay

Adsorption assay was carried out as previously described with minor modifications^10^. In order to quantify the amount of adsorbed virus, cells were infected together with β-escin and AH and incubated for 1 h at 4 °C. The inoculum was then discarded, and cells were washed, supplemented with fresh medium, and incubated for a further 24 h at 37 °C. Virus yields were collected and titrated by plaque assay.

### Penetration assay

Penetration assay was carried out as previously described with minor modifications^10^. To determine the levels of internalized virus, cells were infected and incubated for 1 h at 4 °C. The inoculum was discarded, and cells were washed and treated with β-escin and AH for 2 h at 37 °C. Cells were then washed, and the non-internalized virus was inactivated with citrate buffer (pH 3) for 1 min. Cells were further incubated with fresh medium for 24 h at 37 °C. Virus yields were collected and titrated by plaque assay.

### Immunofluorescence assay (IF)

The procedure for IF was performed as previously described^10^. Cells were fixed with methanol for 10 min at − 20 °C, washed with PBS, and stored in PBS at 4 °C until processing. Cells were subsequently incubated with the primary and secondary antibody for 30 min at 37 °C and then incubated with DAPI. Microscopy and photography data were obtained using an Olympus IX71 fluorescence microscope. The analysis was performed using Fiji software (version 1.53v) (National Institutes of Health, Bethesda, MD, USA).

### Cytokine determination

Mouse IL-6 and TNF-α were analyzed using commercial ELISA sets following the manufacturer's instructions (BD OptEIATM, Becton–Dickinson).

### Transfections and reporter gene assays

The procedure for transfection was performed as previously described^7,8,10^. Lipofectamine 2000 reagent (Invitrogen) was used for transfection assays, following the manufacturer’s instructions. The NF-κB-LUC reporter vector and RSV-β-gal plasmid were provided by Dr. Susana Silberstein (Universidad de Buenos Aires, Argentina). The second reporter control plasmid utilized was RSV-β-gal, encompassing the bacterial β-galactosidase gene regulated by the viral RSV promoter. Luciferase Assay System E1500 (Promega) and β-Galactosidase Enzyme Assay System E2000 (Promega) were used for reporter quantitation following the manufacturer’s instructions.

### Statistical analysis

Statistical analysis was performed as previously described^7,8,10^. One-way analysis of variance (ANOVA) followed by a Tukey’s multiple comparison test was used to assess statistical significance, with the software GraphPad Prism 8.3. p-value < 0.05 was considered statistically significant.

## Results

### Antiviral activity of β‑escin and AH against infection with coronaviruses

**Figure 1:**
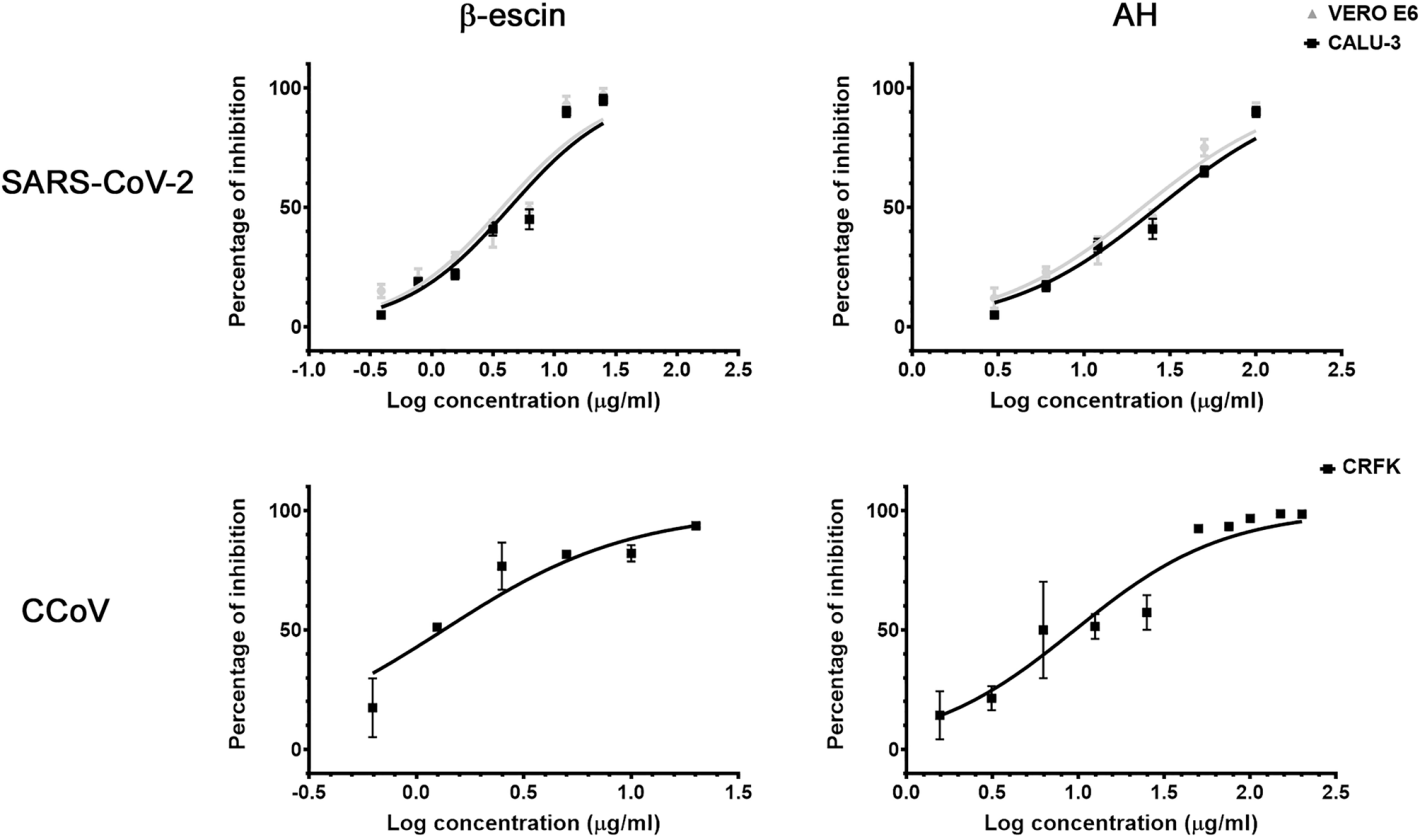
Dose-dependent response of β-escin and AH on viral replication. Antiviral activity of different concentrations of β-escin and AH against CCoV in CRFK cells and SARS-CoV-2 in calu-3 and Vero E6 cells. Data represent mean ± SD for n = 3 independent experiments, performed in triplicate.

In order to examine the antiviral effect of β-escin and AH, we carried out a dose-inhibition assay in infected cells. β-escin and AH significantly reduced viral titers of CCoV and SARS-CoV-2 in a concentration-dependent manner, without affecting cell viability (Table 1 and Fig. 1).

**Table 1:** EC_50_ of β-escin and AH against coronaviruses.

	SARS-CoV-2		CCoV
	VERO E6	CALU-3	CRFK
β-escin	3 ± 0.1	4.3 ± 0.1	1.3 ± 0.4
AH	21.7 ± 0.2	26.7 ± 0.1	9.5 ± 3.8

EC_50_: Effective Concentration 50. EC_50_ (μg/ml) were calculated by nonlinear regression.

Data represent mean ± SD for n = 3 independent experiments, performed in triplicate.

**Figure 2:**
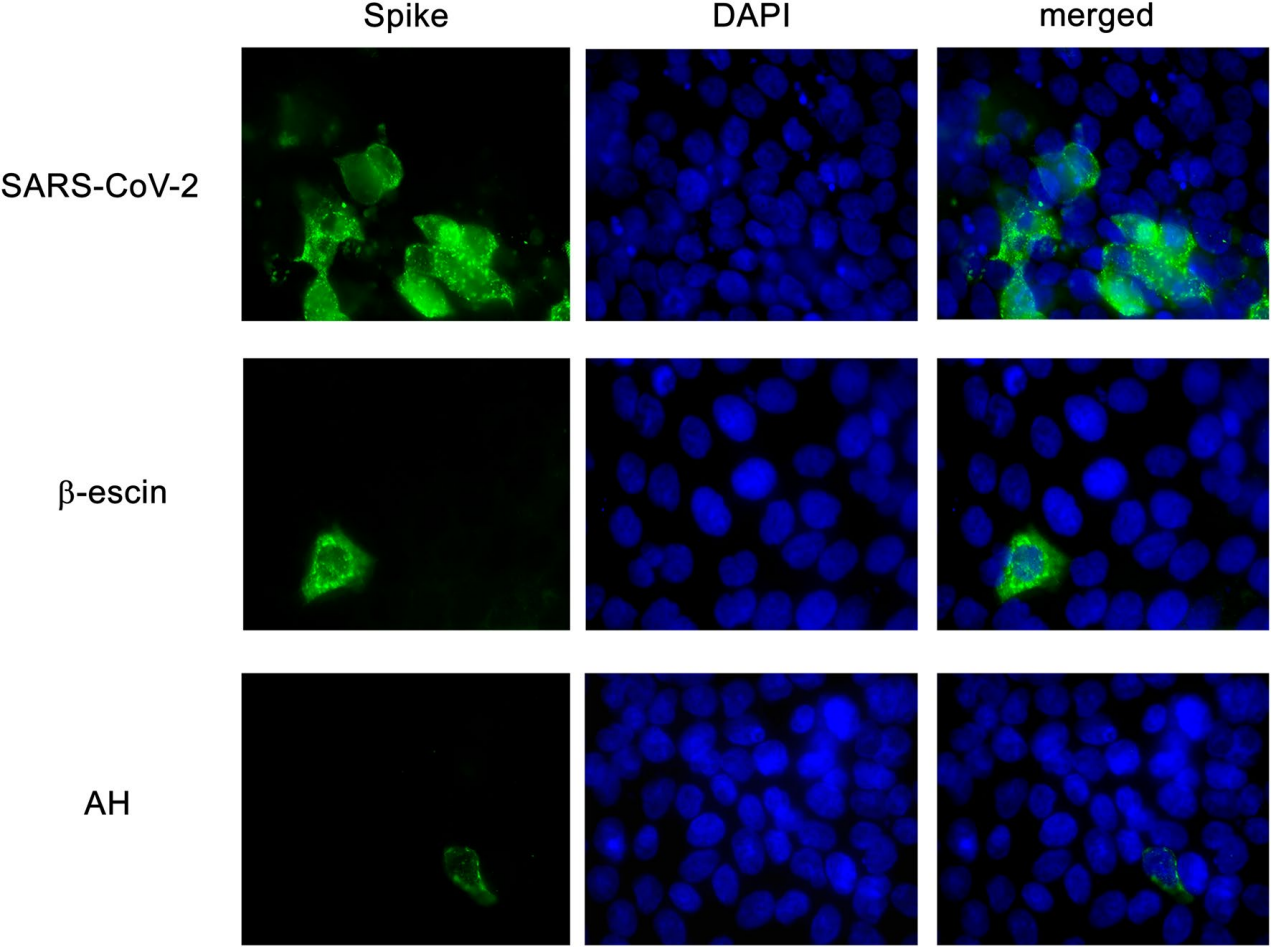
Effect of β-escin and AH on virus protein expression. IF staining was performed to detect the intracellular localization of SARS-CoV-2 spike glycoprotein in calu-3 cells infected with SARS-CoV-2 (moi = 0.1) and treated or not with β-escin (10 µg/ml) and AH (100 μg/ml) at 24 h p.i. Magnification: 400X.

Next, an IF was performed to confirm the inhibitory effects of β-escin and AH on cells infected with SARS-CoV-2. The results indicated that β-escin and AH limited virus infection and reduced SARS-CoV-2 Spike protein expression (Fig. 2). Overall, these findings show that β-escin and AH are effective in inhibiting coronaviruses infection.

**Figure 3:**
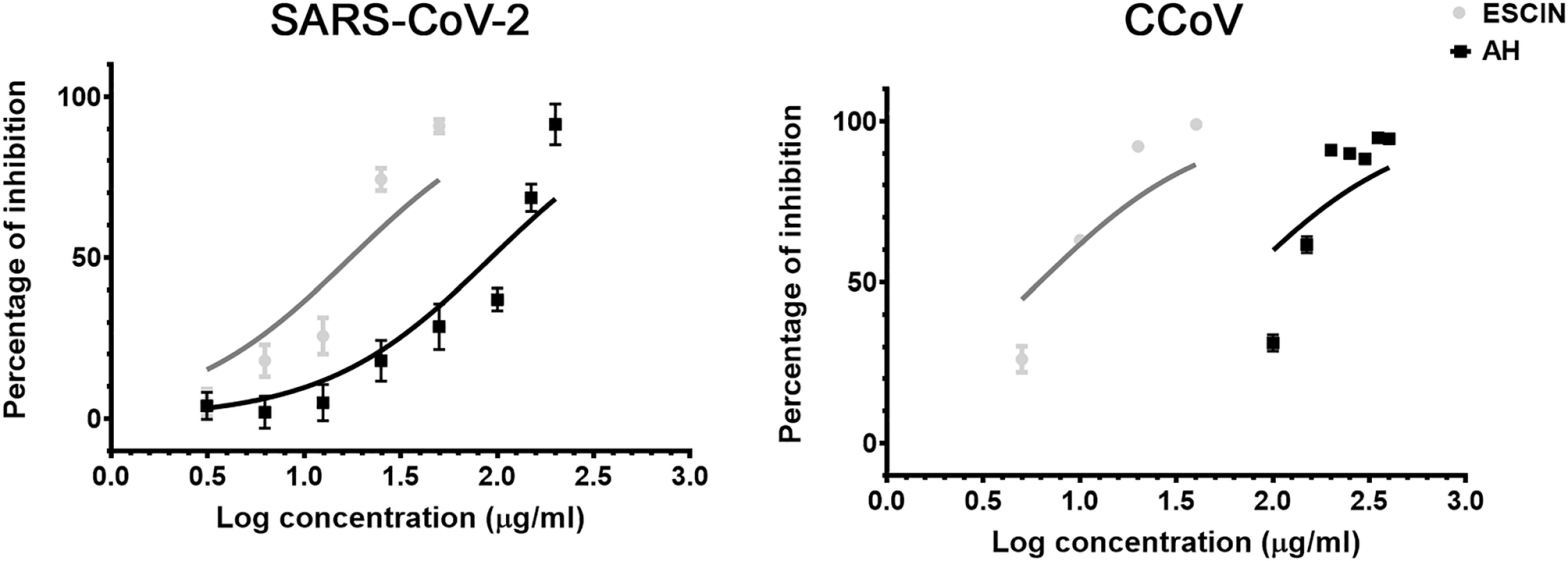
Dose-dependent response of β-escin and AH on viral inactivation. Virucidal activity of different concentrations of β-escin and AH against SARS-CoV-2 and CCoV. Data represent mean ± SD for n = 3 independent experiments, performed in triplicate.

Following this, we used a virucidal assay to determine whether β-escin and AH affect virion stability. The results showed that β-escin and AH reduced SARS-CoV-2 and CCoV titers in the assay (Table 2 and Fig. 3), which further indicates that β-escin and AH can directly reduce coronavirus stability. However, the concentrations of β-escin and AH needed to inactivate coronavirus particles were higher than those observed for antiviral activity, similarly to our previous reporting for RSV and HSV-1^7,8^.

**Table 2:** Virucidal activity of β-escin and AH against coronaviruses.

	SARS-CoV-2	CCoV
β-escin	17.3 ± 1.2	6.1 ± 2.8
AH	92.98 ± 2.4	67 ± 2.7

EC_50_: Effective Concentration 50. EC_50_ (μg/ml) were calculated by nonlinear regression.

Data represent mean ± SD for n = 3 independent experiments, performed in triplicate.

These data demonstrate that β-escin and AH have virucidal and antiviral activities against coronaviruses in vitro*.*

### Influence of the duration of treatment with β-escin and AH on coronaviruses infectivity

**Figure 4:**
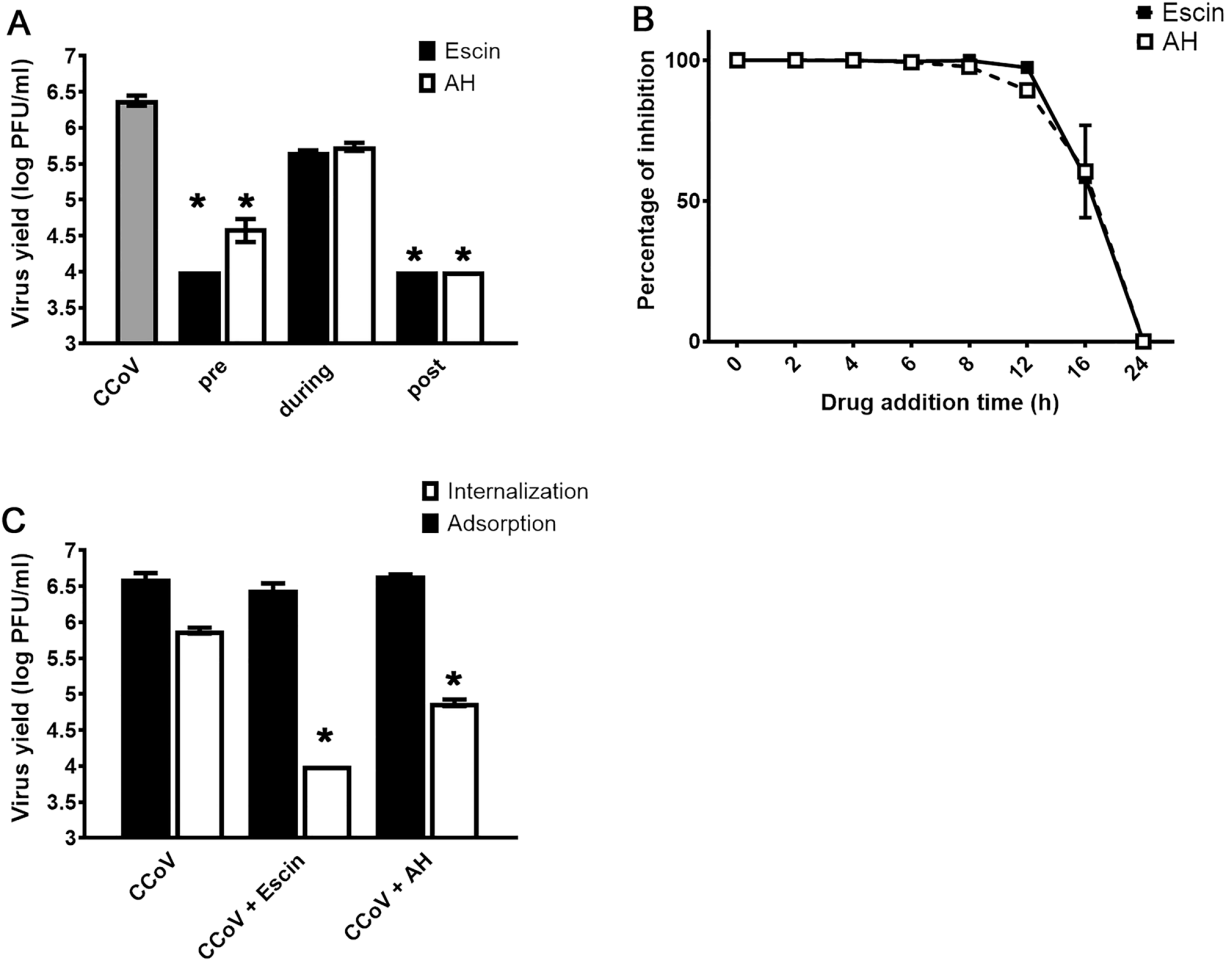
Influence of the duration of treatment with β-escin and AH on CCoV infectivity. (**A**) For pre-treatment assays, cells were treated with the drugs for 2 h at 37 °C, washed with PBS and then infected with CCoV (moi = 0.1). For co-treatment, cells were simultaneously infected with CCoV and treated with the compounds of interest. After 1 h of adsorption at 37 °C, the virus-drug mixture was removed, washed and compounds free medium was added. For post-treatment assays, cells were infected with CCoV for 1 h at 37 °C and then treated with the tested compounds during 24 h. (**B**) Cells were infected with CCoV for 1 h at 37 °C and then treated with the tested compounds at 0, 2, 4, 6, 8, 12 and 16 h after infection. A control culture that was infected but not treated (CV) was simultaneously studied. Cells were further incubated at 37 °C for up to 24 h p.i., and after cell disruption by freezing and thawing, supernatants were titrated by plaque assay in CRFK cells. (**C**) Virus adsorption: CRFK cells were infected with CCoV (moi = 0.1), together with β-escin and of AH and incubated for 1 h at 4 °C. The inoculum was discarded, and cells were washed, supplemented with fresh medium, and further incubated for 24 h at 37 °C. Virus yields were collected and titrated by plaque assay. Virus internalization: CRFK cells were infected with CCoV (moi = 0.1) and incubated for 1 h at 4 °C. The inoculum was discarded, and cells were washed and treated with β-escin and of AH for 2 h at 37 °C. Cells were subsequently washed, and non-internalized virus was inactivated with citrate buffer (pH 3) for 1 min. Cells were further incubated with fresh medium for 24 h at 37 °C. Virus yields were collected and titrated by plaque assay. Data represent mean ± SD for n = 3 independent experiments, performed in duplicate. *Significantly different from CV (p-value < 0.05).

To further characterize how β-escin and AH interfere with the coronavirus life cycle, time-of-addition assays were performed. β-escin and AH were added at different time points relative to the infection with CCoV in CRFK cells: pre-treatment 2 h (pre), co-treatment (during) and post-treatment 1 h (post) (Fig. 4A). Considering that 10 μg/ml of β-escin and 100 μg/ml of AH did not affect virion stability, we selected these concentrations of β-escin and AH in order to describe their antiviral activity.

When β-escin and AH were added during CCoV inoculation, no significant inhibition of viral multiplication was detected. However, CCoV virus yields significantly decreased when β-escin and AH were added before and after infection (Fig. 4A).

Following this, we decided to make a time of addition assay at different times after infection. Results showed that β-escin (10 μg/ml) and AH (100 μg/ml) were able to inhibit infectious particle formation even when both were added at 12 h p.i. (Fig. 4B). At later times, none of the drugs restrained virus infectivity in CRFK cells (Fig. 4B).

To further elucidate the inhibitory effects of β-escin and AH during the earlier stages of the virus cycle, binding and entry assays were performed. The results indicated that β-escin and AH did not exert inhibitory effects during the binding stage. However, the addition of β-escin and AH during virus internalization reduced viral infectivity (Fig. 4C).

Taken together, these findings indicate that β-escin and AH exhibit anti-coronavirus activity by interfering with early stages of the viral life cycle, including blockage of internalization. Furthermore, β-escin and AH exert prophylactic protection against infection with coronaviruses.

### Modulation of NF-κB activation and cytokine production by β-escin and AH in infected epithelial and macrophage cell lines

NF-κB activation following coronavirus infection is necessary for viral replication^11–14^. In addition, we have previously shown that β-escin and AH modulate NF-κB activation in epithelial and macrophage cell lines infected with RSV and stimulated with Toll Like Receptors (TLRs) ligands^8^. As a result, we decided to investigate whether β-escin and AH could also modulate NF-κB activation in epithelial cells infected with coronaviruses.

**Figure 5:**
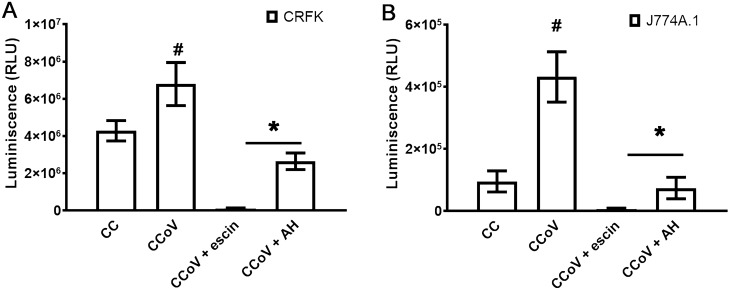
Effect of β-escin and AH on NF-κB activation in CCoV infected cells. (**A**) CRFK and (**B**) J774A.1 cells were transfected with 0.5 μg of NF-κB-LUC reporter vector and 0.5 μg of β-galactosidase control plasmid. After 24 h, cells were infected with CCoV (moi = 0.1) and treated or not with β-escin (10 µg/ml) and AH (100 μg/ml) during 6 h. Luciferase activity was measured in cell extracts, and each value was normalized to β-galactosidase activity in relative luciferase units (RLUs). CC: cell control (unstimulated cells). Data represent mean ± SD for n = 3 independent experiments, performed in duplicate. *Significantly different from CCoV infected cells (p-value < 0.05). # Significantly different from CC (p-value ⋖ 0.05).

As an initial step, we verified that CCoV infection was able to activate NF-κB in CRFK cells. Interestingly, NF-κB signaling pathway was strongly inhibited by β-escin and AH in CRFK cells infected with CCoV (Fig. 5A).

NF-κB activation induced by coronavirus infection not only affects viral multiplication, but also elicits the expression of pro-inflammatory cytokines, including IL-6 and TNF-α, that contribute to inflammation and the pathology of the infection^11–17^. The expression of these cytokines promotes activation and recruitment of immune cells such as macrophages, which resulted in increased secretion of inflammatory cytokines^12,16,17^. Thus, considering the role of macrophages in the inflammatory response against coronaviruses, we decided to analyze the effect of β-escin and AH in macrophages infected with coronaviruses.

**Figure 6:**
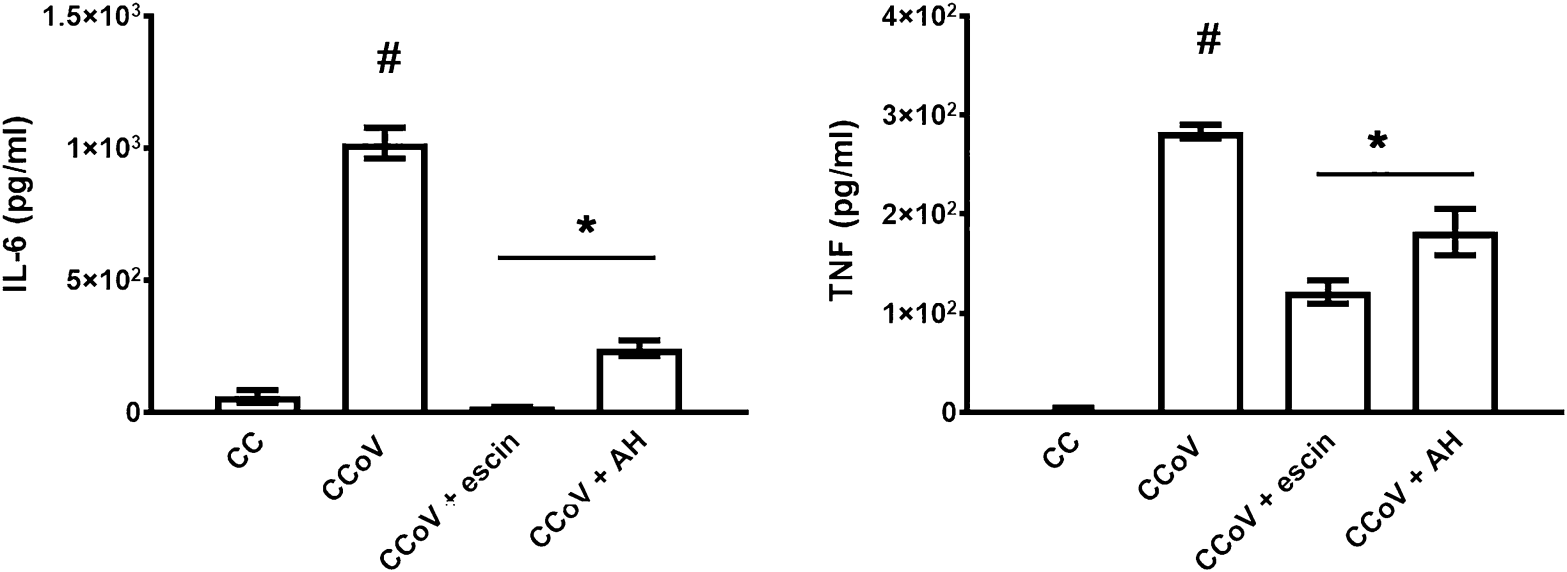
Effect of β-escin and AH on cytokine production in CCoV infected cells. J774A.1 cells were infected with CCoV (moi = 0.1) and treated or not with β-escin (10 μg/ml) and AH (100 µg/ml) during 24 h. IL-6 and TNF-α were determined by ELISA. CC: cell control (unstimulated cells). Data represent mean ± SD for n = 3 independent experiments, performed in triplicate. *Significantly different from CCoV infected cells (p-value < 0.05). # Significantly different from CC (p-value ⋖ 0.05).

We first analyzed whether CCoV multiplies and induces NF-κB activation and cytokine production in J774A.1 cells, a macrophage cell line. We found that CCoV did not productively infect these cells. Nevertheless, CCoV could induce NF-κB activation and cytokine production in J774A.1 cells in the absence of an infection, similarly to results which we have previously reported for RSV^8,10,18^ (Figs. 5B and 6). We then measured the effect of β-escin and AH on NF-κB activation, as well as IL-6 and TNF-α production in CCoV stimulated macrophages. Interestingly, β-escin and AH significantly reduced NF-κB activation and IL-6 and TNF-α production when added to CCoV stimulated J774A.1 cells (Figs. 5B and 6).

In summary, β-escin and AH inhibited the NF-κB signaling pathway in epithelial cells and macrophages infected with CCoV, which could account for the inhibition of viral replication in epithelial cells, and the reduction of IL-6 and TNF-α secretion in CCoV-stimulated macrophages.

## Discussion

Pandemics over the last two centuries have been caused by viruses that include coronaviruses (e.g. SARS-CoV-2) and Influenza (e.g., the H1N1 pandemic of 1918, 1977 and 2009). Thus, considering the probable respiratory virus outbreaks in the future emphasizes the need for broad-spectrum therapies to be prepared for the next pandemics. We must stimulate drug discovery programs for viruses that have the potential to become a pandemic by investing in drugs that function broadly against such virus families. This will enable us to provide protection against new emerging viruses and be prepared to test them in patients when the next pandemic occurs^19^. In addition, acute lower respiratory infections (ALRI), remain a leading cause of morbidity and mortality in children, the elderly, and immunocompromised individuals. Respiratory syncytial virus (RSV), influenza virus, and human metapneumovirus are among the top viral causes of ALRI in children, particularly in low-resource settings^20,21^. Furthermore, SARS-CoV-2 remains a serious problem for patients who are immunocompromised or elderly, despite the fact that hospitalization and mortality rates have decreased since vaccines were introduced^22,23^. In this sense, the World Health Organization advises using antivirals against SARS-CoV-2 to treat COVID-19 patients at high risk of disease progression^24^. In consequence, this calls for the development of drugs in order to combat respiratory virus infections.

Viral infections have long been treated with medicinal plants. Herbal products are ideal candidates for new drugs due to their chemical variety, limited substantial toxic effects, and broad-spectrum antiviral properties^25,26^. In fact, we have previously demonstrated that β-escin and AH exhibit antiviral effect against DNA and RNA enveloped viruses, as well as immunomodulating properties through affecting NF-κB signaling pathway^7,8^. Furthermore, a previous report has shown that compounds with a structure similar to β-escin affect SARS-CoV replication, indicating that they could also have a potential antiviral activity against coronaviruses^27^.

In this study, we showed that β-escin and AH restricted replication of SARS-CoV-2 and CCoV in epithelial cells, demonstrating a broad-spectrum antiviral activity against coronaviruses. The fact that these coronaviruses belong to different genera, *Betacoronavirus* and *Alphacoronavirus*, respectively, support our hypothesis. Importantly, COVID-19 occurs as a result of a primary infection of SARS-CoV-2 in lung epithelial cells, and β-escin and AH inhibited SARS-CoV-2 infection in these cells.

β-escin and AH inhibited coronavirus infection in several aspects, affecting early stages of the viral cycle such as viral internalization. Given their influence on viral replication up to 12 h p.i., it is likely that, in addition to internalization, β-escin and AH may also exert effects on additional viral or cellular targets during the early stages of the viral cycle. This ability to affect multiple targets is what may confer them the characteristic of broad-spectrum antivirals. In this context, considering that the addition of β-escin and AH before infection also affected viral replication, we suggest that they may act on host cells, for instance, by modulating signaling pathways involved in coronavirus replication, such as the NF-κB pathway^11–14^.

SARS-CoV-2 and other coronaviruses trigger NF-κB signaling in epithelial cells and macrophages, which is required for viral replication and cytokine production^11–17^. In consequence of that, NF-κB plays a very important role in the progression of SARS-CoV-2 infection as well as in exacerbating the production of proinflammatory cytokines in the “cytokine storm” observed in patients with severe COVID-19. Thus, the modulation of NF-κB is a promising pharmacological target for COVID-19 treatment, with a low risk of emergence of viral resistance^12,14,16^. We hypothesize that the inhibitory effects of β-escin and AH on coronavirus replication as well as cytokine production in cells infected with CCoV may be associated with the direct downregulation of the NF-κB pathway. In fact, it is proposed that NF-κB activation in coronaviruses infected cells is mediated through the recognition of viral surface proteins by TLR located at the cell surface before viral entry^28–30^. Hence, considering that the activation of NF-κB signaling in response to coronavirus infection is independent of viral entry and replication, β-escin and AH may affect NF-κB activation in coronavirus infected epithelial cells and macrophages, independently of their antiviral activity. In this sense, we have previously reported that β-escin and AH block the activation of the NF-κB pathway and reduce cytokine production in epithelial cells and macrophages that have been stimulated with viral (RSV) and non-viral stimuli (TLRs ligands), independently of viral replication^7,8^.

The viral agents that are currently available are categorized into those that directly inhibit viral replication at the cellular level (antivirals), those that modulate the host response to infection (immunomodulators), and those that directly inactivate infectivity (antibodies or virucides)^31^. We have previously shown that, in addition to their antiviral and immunomodulatory properties, β-escin and AH also exhibit virucidal activity against enveloped viruses, such as HSV-1 and RSV^7,8^. In the present study, we have demonstrated that β-escin and AH also have virucidal properties against coronaviruses. In all cases, β-escin and AH show virucidal properties at higher concentrations that are needed for antiviral activity. This suggests that β-escin and AH may not only be useful as oral antiviral agents, but that they also potentially function as topic microbicidal agents at higher concentrations. Development of virucidal agents for surface and personal hygiene is another important interest, considering their value in preventing and controlling infections caused by SARS-CoV-2 and emerging mutational variants, as well as other future emerging coronaviruses and enveloped viruses (such as Ebola virus, Lassa virus, Nipah virus, and influenza virus)^32^.

Overall, this study demonstrates that β-escin and AH have antiviral and virucidal activities against coronaviruses, as well as NF-κB and cytokine modulating activities in coronaviruses infected cells. Thus, β-escin and AH are promising broad-spectrum antiviral, anti-inflammatory, and virucidal drugs against coronaviruses and respiratory viruses, including SARS-CoV-2. Interestingly, both treatments exhibit similar results, with no differences found in their activities. In view of a clinical application in humans in the future, these biological properties should be examined in animal models of coronavirus infection.

## Data Availability

The data presented in this study are available on request from the corresponding author.
